# Caregiver and care team perspectives of caregiver psychological distress and well-being during critical care hospitalization: a qualitative study

**DOI:** 10.1186/s12877-025-05769-0

**Published:** 2025-03-13

**Authors:** Amanda C. Blok, Lauren Gauntlett, Mayank Jayaram, Sarah L. Krein

**Affiliations:** 1 Center for Clinical Management Research, U.S. Department of Veterans Affairs (VA) Ann Arbor Healthcare System, 2215 Fuller Road, Mail Stop 152, Ann Arbor, MI 48105 USA; 2 Department of Systems, Populations and Leadership, University of Michigan School of Nursing, 400 North Ingalls Building, Ann Arbor, MI 48109 USA; 3 Department of Anesthesiology, The Ohio State University Wexner Medical Center, 410 W. 10th Avenue, Columbus, OH 43210 USA; 4 Department of Internal Medicine, University of Michigan, 1500 E. Medical Center Drive, Ann Arbor, MI 48109 USA

**Keywords:** Critical care, Family caregiver, Psychological distress, Critical illness, Hospitalization

## Abstract

**Background:**

Family caregiver psychological distress during an older adult’s critical care hospitalization can compromise their well-being and ability to function in a supportive role for patient recovery. Understanding factors influencing family caregiver distress and well-being during this period is crucial for developing approaches to support caregiver health. We sought to better understand and compare caregiver and care team member perspectives about factors and strategies that affect psychological distress and well-being among family caregivers during a critical care hospitalization.

**Methods:**

Using a qualitative design, we conducted a directed content analysis of semi-structured interview data collected from 20 family caregivers of Veterans in critical care and 12 care team members at a US Veterans Affairs medical and surgical intensive care unit between October 2020 and July 2021. We examined factors related to caregiver psychological distress or well-being. The Consolidated Criteria for Reporting Qualitative Research guidelines were followed.

**Results:**

Factors identified as related to caregiver psychological distress by caregivers and care team members included unfamiliarity with the health system, care team, and treatment processes; uncertainties about the illness and patient appearance; and responsibilities associated with the caregiver role. Factors related to caregiver well-being included proactive and personal communication, and a comfortable and respectful environment. Within these factors, however, there were differences in focus between caregivers and care teams. Caregivers focused on unfamiliar treatment processes, with unmet expectations around predictable communication. Few care team members indicated awareness of this concern. Other family, home, or caregiving responsibilities were described by caregivers as contributing to distress but were not mentioned by care team members. Caregivers discussed proactive communication by the care team that occurred either in-person or over the phone as emotionally supportive. Care team members emphasized in-person communication and videoconference options as beneficial and comforting to caregivers during visitor restrictions. The impact of a comfortable and respectful environment was recognized as promoting caregiver well-being by primarily non-clinical care team members.

**Conclusions:**

We found parallels between the factors identified by caregivers and care teams related to caregiver psychological distress and well-being, yet often with differences in focus. These findings provide essential information for addressing factors contributing to distress and developing practices that support caregiver well-being.

**Supplementary Information:**

The online version contains supplementary material available at 10.1186/s12877-025-05769-0.

## Background

Older adults are the fastest growing segment of the population to receive critical care in the U.S., and including family in their care is necessary for optimal patient outcomes [1–3]. However, family caregiver psychological distress during critical care is prevalent and often begins at hospitalization, with approximately two-thirds of family caregivers experiencing anxiety during the hospitalization of their family members and in some samples, nine of ten family caregivers reached the level of clinical depression [4, 5]. Family caregivers, also called informal caregivers, are “any relative, partner, friend or neighbor who has a significant personal relationship with, and provides a broad range of assistance for, an older person or an adult with a chronic or disabling condition” [6]. These caregivers often have a central role in the care critically ill older adults receive, including medical decision-making during hospitalization and chronic condition management for survivors in the home setting [7]. However, experiencing psychological harm during a critical care hospitalization can thwart caregiver effectiveness in their role, including impacting their decision-making, and reducing their ability to help manage a patient’s condition after discharge [8–10].

In prior studies, qualitative methods have been used to explore family caregivers critical care hospitalization experiences from either the caregiver’s or care team’s perspective, but rarely both. While studies incorporating both perspectives have examined caregiver participation [11], decision-making [12, 13], information support [14], and visitation [15], as well as clinician behaviors and support while in the intensive care unit (ICU) [16, 17], knowledge of caregiver psychological distress and well-being remains fragmented. To effectively address, and potentially mitigate, caregiver psychological harm, a more complete understanding of factors that influence family caregiver distress during hospitalization is needed. Additionally, while it is known that mental well-being is more than the absence of psychological distress or illness [18], and that caregiver resilience in critical care is inversely related to anxiety and depression [19, 20], factors that promote caregiver well-being during a critical care hospitalization are underexplored.

We sought to understand factors related to caregiver psychological distress and well-being during a patient’s critical care hospitalization through qualitative interviews with caregivers and different types of care team members. Specifically, we aimed to compare caregiver and care team member perspectives of factors related to caregiver psychological distress and well-being during critical care. For this study, we recruited family caregivers of Veterans hospitalized in critical care. In general, hospitalized Veterans tend to be older adults with an average age of 75 years and have multiple comorbid conditions [21]. Understanding caregiver experiences and care team perspectives is an essential first step for addressing modifiable factors contributing to distress and enhancing or further developing practices that support caregiver well-being.

## Methods

### Study design and sample

We used a descriptive qualitative methodology to identify and better understand factors related to psychological distress and well-being among family caregivers during a Veteran patient’s ICU hospitalization. This study took place at a Midwestern tertiary care Veterans Affairs (VA) medical center serving approximately 75,000 Veterans, was approved by the local institutional review board, adhered to the Declaration of Helsinki and followed the Consolidated Criteria for Reporting Qualitative Research (COREQ) reporting guidelines (see Supplementary File 1).

We identified family caregivers through electronic health record (EHR) review. The ICU nurse manager was consulted twice weekly to screen out caregivers who should not be contacted for recruitment (e.g., perceived as potentially inappropriate due to known violence or relational instability), with few exclusions (*N* = 3). Research information letters were mailed to eligible caregivers, followed by phone recruitment. Because this study was conducted during the COVID-19 pandemic, caregiver participants were only recruited by phone due to visitor restrictions. These restrictions fluctuated during the study period as information about the pandemic changed. Nighttime visitation was not allowed for the full study period. Early in the study period, visitors were only allowed if the patient was at the end of life. The policy then changed to allow one or two visitors, in particular essential caregivers (a legal guardian or someone who needed to learn a specific skill at the time of discharge). Recruiting by phone allowed us to reach a variety of caregivers, including some who had limited visitation due to visitation restrictions as well as caregivers who did not have limitations because they were the one visitor the Veteran was allowed on the unit and/or had end of life visitation.

Additionally, we identified nurses, physicians, social workers, respiratory therapists, and physical therapists that authored ICU documentation in the EHR. Potentially eligible staff in roles that do not use the EHR, including unit clerks and chaplains, were identified by the ICU nurse manager. Staff were recruited through email and by phone. Of 39 caregivers and 47 care team members approached, 20 caregivers and 12 care team members participated in interviews between 10/7/2020–7/1/2021. In both groups, reasons for declining participation included lack of time, no interest, or no reason given.

### Data collection

One-time semi-structured interviews were conducted by telephone by a nurse scientist and non-clinician interviewer (AB, LG) using interview guides developed by the study team. The research and guides were based on a combination of theories, including the Self and Family Management Framework, Patient and Family Engagement Framework, and positive psychology [22, 23]. While the management and engagement frameworks focus on how health condition, knowledge and beliefs, healthcare system and team processes, home and clinical environment, and financial and social resources influence patient and family outcomes [24, 25], positive psychology emphasizes optimal functioning in the areas of positive emotion, engagement, meaning, positive relationships, and accomplishment [23, 26]. Caregiver interviews concentrated on caregiver experiences during the ICU hospitalization. Care team member interviews focused on family engagement and distress during critical care, including current practices and opportunities to provide family support. All interviews were audio recorded and transcribed verbatim.

### Data analysis

We conducted a directed content analysis [27]. We developed a codebook using the previously identified theoretical frameworks. Two study team members (AB, LG) independently coded seven family caregiver interview transcripts and met to compare coding and reconcile discrepancies through consensus. The remaining transcripts were divided and coded separately. The same process was used with care team member interview transcripts. NVivo 12 software was used to generate code reports for each group, which were initially analyzed separately. For this analysis, we identified factors specifically mentioned or attributed to caregivers feeling distressed, and the context when participants used terms of psychological distress (e.g., stress, depression, anxiety, trauma). Similarly, we identified factors that contributed to family caregiver well-being by identifying positive expressions and emotions, such as comforting and helpful. A matrix approach was used to explore similarities and differences between caregiver and care team member responses. We have written summaries of these findings below, including providing illustrative quotes from participants in the text and tables. Ongoing discussion, as well as searching for disconfirming cases and reconciliation of coding discrepancies, were used to establish rigor.

## Results

Twenty family caregivers and 12 care team members were interviewed. Participating caregivers included the patient’s significant other [11], child [3], parent [3], sibling [2], and one other family relationship [1]. Caregiver interviews lasted an average of 48 min (range 26–61 min). Care team member interviews were conducted with four providers (pulmonologist, intensivist, resident, physician assistant), three registered nurses, three ancillary staff (a respiratory therapist, a physical therapist, a social worker) and two non-clinical staff (a cleark and a chaplain). Participant experience providing critical care in VA ranged from one to ten years (average of four years). Clinician interviews lasted an average of 47 min (range 26–59 min).

Among the factors identified by caregivers and care team members as contributing to psychological distress during critical care were unfamiliar health care and treatment processes, illness uncertainty and patient appearance, and responsibilities related to the caregiver role. Factors identified as supporting caregiver well-being included proactive and personal communication, and a welcoming and respectful environment. While the factors identified by caregivers and care team members were generally similar, there were notable differences between groups.

### Factors related to caregiver psychological distress during critical care

#### Unfamiliar health care and treatment processes

Caregivers often discussed distress related to a lack of familiarity with the healthcare system and treatment processes, including care team communication and decision-making practices. In contrast, care team members rarely mentioned these factors as potentially causing distress (Table 1). For example, caregivers reported distress with not knowing when the care team would be in contact with treatment or condition updates.

*“I did not have the doctor come out to me afterwards*,* so that was stressful right away*,* I mean I was stressed the whole day but you know*,* worrying about the surgery*,* but that added to it” (Spouse of Veteran*,* Caregiver 04)*.

Some caregivers also expected to be informed in advance about medication and treatment changes, and were distressed when treatments were not clearly communicated.

*“I mean I didn’t really know what they were doing and it made me quite nervous… there was just so much switching pills around… I wonder if these doctors are keeping tabs on each other.” (Sibling of Veteran*,* Caregiver 13)*.

Although care team members rarely mentioned these factors as contributing to caregiver distress, one team member acknowledged caregiver anxiety while waiting for information during what they characterized as the “initial phases of getting on the unit.” Care team reported strategies to address unfamiliar health care processes were also rare, although one provider discussed the potential benefit of a brief patient and caregiver orientation to the unit prior to a planned surgical procedure that would require subsequent critical care.

**Table 1:** Unfamiliar health care and treatment processes related to caregiver psychological distress during critical care

Illustrative quotes from caregivers (*N* = 20)	Illustrative quotes from care team members (*N* = 12)
“I mean that was a worrisome time, wondering and when we were going to get the answer…” (*Spouse*,* Caregiver 01*) “The scariest thing is when they said they might put him on a ventilator and I thought I didn’t even know how, didn’t have any idea how that goes or how it works, maybe if they would’ve explained that a little bit more” (*Significant other*,* Caregiver 15*) “Every time they come in, they’ll ask him, “What medicines do you take?” I mean it’s already in the chart, I mean I don’t understand…they just keep asking him all these questions over and over again” (*Spouse*,* Caregiver 18*)	*Sole factor quote*:^*a*^ “I think, you know perioperatively when they’re in the OR, there’s a lot of anxiety that seems to come across because they’re not aware of you know how much longer, are they doing okay, and so they come in a little bit worried, like, “Oh my gosh, I’ve been waiting out there to find out if he’s okay for some time,” and you know I think it’s just the initial phases of getting into the unit” (*Provider*,* Team member 11*) *Strategy quote*:^*b*^ “If I know that they have a super anxious spouse or something at home, if I find you know something that’s changed or something I know that that spouse was concerned about…, then I would definitely update the family on that.” (*Nurse*,* Team member 09*) *Strategy quote*: “In advance of the actual surgical date, assuming it was not an emergency or an unanticipated surgery…would be the right time is to have the patient you know and family members sort of prepare themselves mentally for what could potentially be kind of a rocky period.” (*Provider*,* Team member 07*)

**Notes.**^a^ Care team members provided insight on factors that related to family caregiver psychological distress, and we include some illustrative quotes in our tables. Because this quote is the only quote from a care team member around unfamiliar health care and treatment processes related to caregiver psychological distress, we have provided a sublabel “Sole factor quote” to emphasize this point

^b^ Care team members also provided strategies they use to address these factors, and we include illustrative quotes around these in our tables

#### Uncertainty about the illness and patient appearance

Caregivers expressed distress around the uncertainty of patient illness and unexpected changes (Table 2). Some caregivers described experiencing stress whenever they received a medical update from providers over the phone because of the unknown nature and fear of bad news, yet were glad to receive the information.

*“it was tough because you didn’t know what the phone call was going to be*,* but I’m glad that they called each time they had to.” (Child of Veteran*,* Caregiver 16)*.

Care team members were cognizant of caregiver concerns about different aspects of the patient’s condition and in some situations tailored communication to try to mitigate caregiver distress.

*“It’s sort of on a case-by-case basis how in-depth I will go with someone because some people really want to know everything… some people just want a very thin explanation of what’s going on and anything else seems overwhelming to them.” (Nurse*,* Team member 12)*.

Both caregivers and care team members also described patient appearance as a source of shock and potential trauma.

*“He obviously was attached to a million things and so it was scary” (Spouse*,* Caregiver 03)*.

However, care team members also recognized the value of caregivers seeing the patient to improve understanding of the patient’s condition severity and trajectory, and better prepare caregivers for difficult end of life decisions.

*“I think [the family seeing the patient] would be better for me because I’m not dropping a bomb on somebody… because*,* you know I hate to say it*,* but I feel like doctors sometimes aren’t like extremely forthcoming with how serious certain situations are and I think trends speak to people and so if someone can see that this [the patient’s condition] is continually getting worse*,* or this is continually getting better*,* I think that would comfort or reduce their anxiety at least.” (Provider*,* Team member 11)*.

Care team member strategies for supporting caregivers in relation to illness uncertainly and patient appearance included determining caregiver understanding through conversation and questioning, inviting participation in medical rounds, as well as listening and validation of caregivers’ emotional reactions.

**Table 2:** Uncertainties around patient illness and appearance related to Caregiver Psychological distress during critical care

Illustrative quotes from caregivers (*N* = 20)	Illustrative quotes from care team members (*N* = 12)
“We were just, almost kind of in shock knowing what was coming up… and I didn’t actually know anything at the end of surgery whether you know, the pathology” (*Spouse*,* Caregiver 01*) “The minute I heard the kidneys were failing and he had to have dialysis, that just threw me for a loop because I wasn’t expecting that at all” (*Sibling*,* Caregiver 13)* “when you go one day and they seem to be getting better and then you go the next day and they seem to be regressing, then it’s kind of worrisome.” (*Fiancé*,* Caregiver 05*) “It was a very scary situation… have him laying on the bed in excruciating pain was kind of scary” (*Fiancé*,* Caregiver 05*)	*Factor quote*:^*a*^ “if a patient was on room air or was just on a nasal cannula and then we have the need to intubate them, that of course we would call a family member for just because that, like being restrained, that can be a very shocking thing to see and so we do, we do update them you know if we’re intubating or doing anything major like that, putting in lines, you know like arterial pressure lines or central lines, we always let them know.” (*Nurse*,* Team member 06*) *Factor quote*: “I mean it’s with Critical Care you know some things can be very uncertain. I think some things can really be traumatic for family members. Some of the things that patients are connected to, how patients are doing” (*Ancillary staff*,* Team member 02*) *Strategy quote*:^*b*^ “I do a lot of just listening and you know letting them know it’s okay, it’s okay to have whatever emotion that they’re having. So you know sometimes they can be angry and that’s okay. They’re going through stressors, they’re seeing things that they haven’t seen before and that’s okay and you can talk to me about it.” (*Ancillary staff*,* Team member 02*) *Strategy quote*: “personally I always ask like the patient and the family if they’re around, like do you want, do you have any questions for the physicians, we’re going to do rounds, you know feel free to listen or participate as much or as little as you want, you know I just make sure to make them feel welcome or try to” (*Nurse*,* Team member 12*)

**Notes**. ^a^ Care team members provided insight on factors that related to family caregiver psychological distress, and we include some illustrative quotes in our tables

^b^ Care team members also provided strategies they use to address these factors, and we include illustrative quotes around these in our tables

#### Responsibilities related to the caregiver role

Several caregivers identified distress related to their role as a caregiver. This included having to update family members with medical information, not knowing how to assist the patient during the hospitalization or after discharge, and dealing with other family obligations (Table 3).

Interpersonal dynamics, as well as new responsibilities that made it difficult to be present at the hospital, such as taking care of the patient’s dependents, were sources of stress for some caregivers.

*“[the patient] kind of got grumpy with me and told me I was useless*,* I might as well go home…” (Spouse to Veteran*,* Caregiver 03)*.

*“It’s horrible*,* because you don’t know what to expect! You don’t know what [the patient is] supposed to do*,* what they’re not supposed to do*,* what they’re supposed to eat…” (Parent to Veteran*,* Caregiver 14)*.

Care team members were cognizant of the potential psychological impact related to the caregiver role but were primarily focused on distress due to family visitation policies that prohibited caregivers from visiting units during the COVID-19 pandemic.

*“Almost everyone’s emotional right now*,* almost everyone that I speak to is scared and they don’t understand what’s going on and they miss their loved one… I get the sense that that’s probably the biggest fear that they have aside from losing their loved one*,* is that their loved one is there and alone and afraid.” (Nurse*,* Team Member 06)*.

Care team members supported caregivers during visitation bans by communicating empathy, drawing on personal experience, letting caregivers know their messages are important and relayed to the patient.

**Table 3:** Responsibilities of the caregiver role related to caregiver psychological distress during critical care

Illustrative quotes from caregivers (*N* = 20)	Illustrative quotes from care team members (*N* = 12)
“which is stressful too because you’re just going over and over everything, but you need to update certain people” (*Spouse*,* Caregiver 04*) “I mean it’s really hard when somebody is in the hospital and you’ve got a try to take care of a home and visit and everything, you know, and then I felt guilty if I didn’t go.” (*Spouse*,* Caregiver 18*) “I’m almost 70 and I’m dealing with his almost six-year-old son at the same time” (*Parent*,* Caregiver 14*) “When he went into the hospital, then I just, there was nothing to do here for him, I just, you know, just worry about him and go see him and that was about it.” (*Sibling*,* Caregiver 13*) “I had so much on my mind… he’s [the patient] always saying that I’m the problem. No, I’m not, you know.” (*Parent*,* Caregiver 14*)	*Factor quote*:^*a*^ “if you’re in a sustainable caregiver role or even a challenging caregiver role, there’s mental health concerns that can come with that….” (*Provider*,* Team member 01*) *Strategy quote*:^*b*^ “I always encourage families to actually record on their phone while they’re getting the training as long as everyone’s okay with that and consents to that, because I often know families are overwhelmed and they’re going to get home with the patient and be like, ‘I have no idea what I’m supposed to do’” (*Ancillary staff*,* Team member 04*) *Strategy quote*: “So, I do think there’s, there’s some benefit in being exposed [to care activities during hospitalization] and trying to get a better understanding.” (*Provider*,* Team member 01*) *Strategy quote*: “it’s just you know letting them know basically that I understand and that I’m not just a nurse, I’ve been a patient or a patient’s family myself and I get it.” (*Nurse*,* Team member 06*)

**Notes**. ^a^ Care team members provided insight on factors that related to family caregiver psychological distress, and we include some illustrative quotes in our tables

^b^ Care team members also provided strategies they use to address these factors, and we include illustrative quotes around these in our tables

### Factors perceived as promoting caregiver well-being during critical care

#### Proactive and personal communication

For caregivers and care team members proactive information sharing was the most important factor in promoting caregiver well-being during critical care (Table 4). Caregivers felt most supported when information about the patient was communicated by care team members proactively, in a personal manner and when questions were elicited. Additionally, emotional support in the form of comforting statements, respect, personal sharing, genuine excitement with patient improvement, kindness and humor provided emotional relief for families.

*“the staff*,* whether it was the doctors*,* the nurse*,* whether it was the guy mopping the floor… communicated a tremendous amount of respect for my dad…they always talked to him*,* and they talked about personal things…that’s the kind of stuff that they don’t necessarily have to do*,* but it’s stuff that meant a lot.” (Child of Veteran*,* Caregiver 16)*.

Likewise, care team members described how they proactively shared information to help caregivers feel a sense of control and attempted to include caregivers by inviting them to listen or participate in care team activities and setting goals.

*“I obviously like always update and educate regularly while I’m giving care to the Veteran and try to involve the family member as much as possible because I think I can help them feel little bit more of a sense of control you know when their loved one is ill.” (Nurse*,* Team member 12)*.

Although phone calls were to some degree a source of distress, caregivers also discussed feeling relieved by this type of outreach and communication by the care team. Care team members, on the other hand, also recognized the benefits of in-person communication, or during pandemic-related visitor restrictions, the use of video tablets that allowed family to see patients being cared for as a way of providing comfort.

*“I have to tell you*,* it [getting calls at home from hospital] was very heartwarming*,* but also gave me a sense of a little bit of relief knowing that if I wasn’t there*,* they’re still and hands-on taking care of things at the immediate moment.” (Child of Veteran*,* Caregiver 02)*.

*“Generally families don’t need us as much when they’re able to come and sit with the patient and be there while all the doctors and specialty providers are coming in because then they just feel like they know what’s going on… I think helps them feel in control.”(Ancillary staff*,* Team member 04)*.

**Table 4:** Proactive and personal communication related to caregiver well-being during critical care

Illustrative quotes from caregivers (*N* = 20)	Illustrative quotes from care team members (*N* = 12)
“The doctor was really super, he was very comforting to me, he assured me you know that they were to do everything they could, he told me not to worry… it was a horrible experience and yet I feel like you guys made it workable, so I could get through it.” (*Parent*,* Caregiver 12)* “they had to catheterize him and it was not pleasant for him but you know, he knew that that had to be done and they explained that to us very well and assured us that, you know, it was going to help and it did. I mean, it was tremendous.” (*Spouse*,* Caregiver 09*) “I think the fact that the doctor called me personally and let me know what was going on … he was very patient with me on the phone so that I could ask all the questions that I had. I think that helped tremendously.” (*Significant other*,* Caregiver 21*) “He had a nurse in particular that would explain, that would come in the room after everybody, because if a group of interns came in, when they left, he would come back and ask me did I get what they were trying to say.” (*Fiancé*,* Caregiver 05*) “I have to tell you, it [getting calls at home from hospital] was very heartwarming, but also gave me a sense of a little bit of relief knowing that if I wasn’t there, they’re still and hands-on taking care of things at the immediate moment.” (*Child*,* Caregiver 02*)	*Factor quote*:^*a*^ “I feel like [including caregivers] gives comfort and I feel like it helps …the patient and the family and for all of us to come together to actually have realistic goals that are meaningful to the patient.” (*Physical therapist*,* Team member 02)* *Factor quote*: “The video tablets were definitely a comfort to the patients and the family… more so than a telephone, just to be able to see their face provides even, so much more comfort… family seeing the patient and knowing that they’re, you know, they’re being cared for.” (*Physician assistant*,* Team member 11*)

**Notes**. ^a^ Care team members provided insight on factors that related to family caregiver well-being, and we include some illustrative quotes in our tables

#### Comfortable and respectful environment

Caregivers were reassured when the environment felt calm and respectful, leading to feelings of comfort and safety for the patient (Table 5).

*“I was okay*,* I felt very comfortable*,* very at peace. You know both rooms*,* the recovery room*,* for a recovery room it was busy and it really was quiet*,* you know what I mean? Everybody kind of respected everybody you know…” (Spouse of Veteran*,* Caregiver 11)*.

Care team members rarely mentioned environmental factors in relation to caregiver well-being, with a few notable exceptions. A clerk described how hospital-based amenities provide caregivers physical comfort and an opportunity to “gather themselves” or take their mind off of the patient’s critical illness. A physical therapist also described how they prepare the patient’s environment and position to support interaction between the patient and caregiver.

*“Usually the first time it’s nice to have kind of the patient’s family come in after they’re [the patient is] up in a chair and just kind of see them be a little bit normal*,* you know have their table in front of them*,* help them engage*,* so that’s the*,* the biggest key moment… when the patient starts to engage.” (Ancillary staff*,* Team member 02).*

**Table 5:** Comfortable and respectful environment related to caregiver well-being during critical care

Illustrative quotes from caregivers (*N* = 20)	Illustrative quotes from care team members (*N* = 12)
“When I went up, the room was really nice and it was huge, I couldn’t believe how big his room was and I just I felt safe knowing that he was there. I felt good about his safety.” (*Sibling*,* Caregiver 13*) “They [care team] would advise me that you know, “You don’t have to stay here all day, you could go home and take a nap if you want to come back, make sure that you’re eating,” you know they would always make sure to tell me take care of yourself because you’re going to have to be taking care of him later, so you’ve got to be up to this, you know you’ve got to be up for this but yeah, they were always very kind to me…I think it was very helpful and it would, when I would go home I always felt very comfortable leaving him.” (*Significant other*,* Caregiver 21)*	*One of few factor quotes*:^*a*^ “The cafeteria being able to get hot plate…those are little minor comforts that I’ve seen and feel that have helped the family members get through those, those moments… for no other reason just for a different area of the hospital for them to be able to escape to for a moment and gather themselves.” (*Non-clinical staff*,* Team member 05*) *One of few factor quotes*: “Many times the family member will come in and they’ll watch the movie with [the patient] and just be there with them…I feel that that technology [TV, audiobook] there has been helpful for them to be able to get through whatever’s going on, get their mind off of the situation for those brief moments.” (*Non-clinical staff*,* Team member 05*)

**Notes**. ^a^ Care team members provided insight on factors that related to family caregiver well-being, and we include some illustrative quotes in our tables. Because these quotes are only several of the few quotes from a care team member around a comfortable and respectful environment related to caregiver well-being, we have provided a sublabel “One of few factor quotes” to emphasize this point

## Discussion

In our descriptive qualitative study, we identified and compared factors described by caregivers and care team members related to caregiver psychological distress and well-being during a critical care hospitalization. Caregivers and critical care team members both described uncertainty about illness and patient appearance in relation to psychological distress, and proactive and personal communication as impacting caregiver well-being. On the other hand, we found differences in focus and, at times, mismatching perspectives between caregivers and care team members involving other factors. While caregivers focused on unfamiliar health care and treatment processes, with unmet expectations around predictable communication contributing to psychological distress, few care team members indicated an awareness of this concern.

Intensive care settings and processes are unfamiliar and distressing to caregivers [28], yet their impact is largely unrecognized by care team members likely due to their own familiarity. Paper leaflets about the ICU setting can significantly lessen caregiver psychological distress alone [29] or in conjunction with a proactive family conference [30]. How often this relatively simple and low-cost intervention is used is unknown [31]. Care team member lack of awareness of distress caused by unfamiliarity may be a barrier to dissemination and additional strategies for providing family-centered care in the ICU setting are still needed [32].

Caregiver uncertainty about patient illness and treatment has been explored in the adult critical care caregiver population, showing that poor access to information contributes to insecurity and fear [33, 34]. Our care team members described the benefits of in-person communication with caregivers, including seeing the patient to have a shared understanding of the patient’s medical course and facilitate end of life decisions. In-person communication interventions—such as a communication facilitator to support family self-efficacy [35] or a nurse present in physician-led family conferences to support communication [36] —that address caregiver concerns related to illness, medical care and decision-making are moderately effective in reducing distress. Additionally, care team members welcomed family presence in the ICU, including participation in rounds and during treatments, to promote medical knowledge, awareness and a sense of team. These actions are known to support caregiver understanding and involvement in decision making [37, 38], yet are commonly clinician-centered—involving family in what is already occurring. Family-centered interventions that predictably engage family caregivers early on and over time [17], and not only while present, also need to be developed and implemented due to caregiver competing roles and demands.

Caregiver responsibilities, family dynamics and role burden during the critical care period are under-explored in the literature [9, 28] and appear to be under-addressed by care team members. Both caregivers and care team members were distressed by restricted family visitation during the pandemic, yet care team members reported video visits were a new strategy employed to improve patient-caregiver connection. Caregivers unable to visit due to organizational or personal barriers could benefit routinely by teleconference visitation integrated into unit culture.

Personal and proactive communication influences caregiver coping and trust, promoting their well-being and meeting some emotional needs [39, 40]. Prior research indicates a lack of trust in the care given leads to caregivers needing physical closeness to the hospitalized patient [34]. We found proactive communication by care team members brought a sense of emotional relief and helped caregivers to feel comfortable enough to leave the ICU setting. Proactive communication interventions for family to further their well-being—whether they are present or not—should be considered, such as formalizing care team communication with family organized by tools such as note templates within the EHR and providing accessible communication options, such as teleconferenced rounds [38, 41].

While intensive care unit climate and staff-related factors are associated with family well-being [42], there is little descriptive detail around how environmental factors influence family well-being. We found the physical space of the patient room, unit and hospital amenities, as well as respectful or calming behavior and communication by all staff members—including non-clinical staff—can promote a sense of well-being. Our inclusion of care team members, such as a chaplain, clerk and physical therapist, greatly contributed to our understanding of this relationship and strategies to promote caregiver well-being. Notably, a recent randomized trial of chaplains providing proactive spiritual care in the ICU significantly reduced caregiver anxiety measured six to eight weeks after discharge compared to usual care [43]. Pursuing inclusion of a broader spectrum of care team members in the development of interventions to promote caregiver well-being, and mitigate distress, is needed.

There are some limitations with this study. We conducted interviews with caregivers of Veterans hospitalized in a medical-surgical critical care unit at a single tertiary VA medical center, impacting generalizability. While the Veteran population has been found to have greater disability and lower incomes than the general population, this study did not directly assess these factors. Further, the Veteran population is similar to the male Medicare population [44]. Future research could sample caregivers from multiple organizations of both high- and low-resourced settings for a diversity of experiences related to the health system, and potentially identify additional factors for this caregiver population.

## Conclusions

This study provides a broader understanding of the factors influencing caregiver psychological distress and well-being during the period of critical care. Factors related to caregiver psychological distress identified by caregivers and care team members included unfamiliar health system, care team and treatment processes; uncertainties related to illness and patient appearance; and responsibilities associated with the caregiver role. Likewise, factors related to caregiver well-being included proactive and personal communication, and a comfortable, respectful environment. Several distinct differences between caregiver and care team perspectives also emerged, with some of the factors identified by caregivers rarely described by care team members. These findings, along with supportive strategies identified by care team members, provide a basis for developing interventions for caregivers to reduce psychological distress and promote well-being during critical care.

## Electronic supplementary material

Below is the link to the electronic supplementary material.

Supplementary Material 1

## Data Availability

The datasets used and analyzed during this study are available from the corresponding author on reasonable request. All methods were performed in accordance with the Consolidated Criteria for Reporting Qualitative Research guidelines.

## Abbreviations

COREQ: Consolidated Criteria for Reporting Qualitative Research
ICU: Intensive Care Unit
VA: U.S. Department of Veterans Affairs
